# A noninteger order SEITR dynamical model for TB

**DOI:** 10.1186/s13662-022-03700-0

**Published:** 2022-03-26

**Authors:** Jitendra Panchal, Falguni Acharya, Kanan Joshi

**Affiliations:** Department of Applied Sciences and Humanities, Parul Institute of Engineering and Technology, Parul University, Vadodara, Gujarat India

**Keywords:** 34A08, 93A30, 37M05, 26A33, Noninteger order derivative, Numerical simulation, Mycobacterium tuberculosis bacteria, Mathematical model, Dynamical system

## Abstract

This research paper designs the noninteger order SEITR dynamical model in the Caputo sense for tuberculosis. The authors of the article have classified the infection compartment into four different compartments such as newly infected unrecognized individuals, diagnosed patients, highly infected patients, and patients with delays in treatment which provide better detail of the TB infection dynamic. We estimate the model parameters using the least square curve fitting and demonstrate that the proposed model provides a good fit to tuberculosis confirmed cases of India from the year 2000 to 2020. Further, we compute the basic reproduction number as $\Re _{0} \approx 1.73$ of the model using the next-generation matrix method and the model equilibria. The existence and uniqueness of the approximate solution for the SEITR model is validated using the generalized Adams–Bashforth–Moulton method. The graphical representation of the fractional order model is given to validate the result using the numerical simulation. We conclude that the fractional order model is more realistic than the classical integer order model and provide more detailed information about the real data of the TB disease dynamics.

## Introduction

Tuberculosis is a disease instigated by Mycobacterium tuberculosis bacteria (MTB), which commonly affects the lungs; however, it can also harm other parts of the body. It is an infectious disease; around ten percent of the latent infection develops into a highly infected disease and results in death. The transmission of TB from one person to another occurs through air droplets when an infectious person sneezes or coughs [2]. Generally, indications include fever, feeling of coldness, sweats at night, lost appetite, loss of weight, and weakness [2]. Tuberculosis has existed in the world for ages, which is apparent from the Egyptian mummy stored in the British Museum that reveals the infection in its spine due to tuberculosis; however, in the nineteenth and early twentieth centuries, the threat of prevalence in public increased, especially among urban [2]. Visibly the number of cases eventually degraded since the year 2000 [6]. By the year 2018, one-fourth population of the world was assumed to have a latent infection of tuberculosis, and there were around ten million active cases with one and half million deaths in the same year. This history ranks TB as the number one infectious disease causing death [6]. Eighty percent of the positive tested patients are from Asia and Africa, whereas only five to ten percent are tested positive in the USA by the tuberculin test [28]. For five decades, rampant and efficacious treatment for actively infected and inertly infected people has been made accessible. Medicines like Streptomycin combined with Pyrazinamide have been used to cure TB, whereas the two most impactful medications believed to combat Mycobacterium tuberculosis are Isoniazid and Rifampin [39].

In the wake of the COVID-19 pandemic, India has observed a drop in detecting new TB cases by 1.3 million from 2019 to 2020 [1]. Additionally, according to the global 2021 tuberculosis report released by WHO, the number of deaths due to TB has increased thereafter, ranking India among the top countries contributing towards the deduction of diagnosis of TB during the COVID-19 crisis [1]. Approximately 4.1 million undiagnosed individuals are suffering from TB presently. Due to lockdown, the number of people visiting for treatments has also fallen notably [1]. India shared 34% of the total predicted 1.48 million deaths occurred by TB globally, whereas it surged to 13% within the country as compared to 2019 [1].

Mathematical modelling for infectious diseases has been developed and studied thoroughly by many researchers for decades now. In comparison to the traditional integer-order models, the fractional-order models offer more precise and in-depth knowledge related to the complex patterns of numerous diseases owing to their inherited properties and explanation of memory [10, 27, 38]. For instance, Khan et al. [25] developed a novel fractional model for TB, [37] analysed the tuberculosis–HIV system, and [20] discussed a fractional model for fever named dengue. Furthermore, [17] developed a fractional order discrete differential model with a time delay to study the effects of endogenetic and exogenic recurrence in the qualitative behavior of tuberculosis. Moreover, Yang explained the impacts of being dormant for a long time and numerous contagions in the changing patterns of tuberculosis [51]. Bowong [14] examined the slow-moving and speedy evolution of the TB model that includes slow and fast development. The conclusion was if the number of elementary reproductions is smaller than one, the steady-state of disease-free is constant in contradiction to when it is larger than one. The optimal control problem for fractional TB disease model incorporating the effect of diabetes and stress was studied in [42]; besides, its analysis using Atangana–Baleanu derivative was carried out in [10, 45].

The evolution of TB was studied mathematically using epidemiological modeling [13, 21] for a long time; however, Waaler bagged the initial effort to model the disease epidemiology of TB [48]. Later, Kermack and McKendrick bifurcated various stages of disease into diverse compartments, which were originated by [24] and expounded by Baile [33], Anderson and May [9]. In [46], the C-F fractional-order derivative is applied to analyze the TB model. Agusto and Cook analyzed a deterministic model to study transmission after separation, cases that forgot to turn up for checkups, and cases with drug resistance [7]. Melsew, Adekunle, and Cheng worked to prove the presence of widespread heterogeneity among infections of people with active TB [31].

Furthermore, researchers developed mathematical models to study the treatments and ways of cure like vaccination. The mathematical model in [32] demonstrates the efficiency of the vaccination to cure an infection of TB. Egonmwan et al. [36] established a new-fangled TB model to inspect the effect of diagnosis and treatment for the infectious mass of TB. The study in [12] shows that diagnosis, proper vaccination drives, and treatments can restrain uncertain aspects of TB; however [40] mentions that even deficient vaccination of tuberculosis plays a role in inhibiting the spread of the disease. Robert studied a TB system with the consequence of reversion of infection [49].

Some authors have also analyzed the relation between TB and coronavirus looking forward to the recent pandemic. The interpretations given by Marimuthu, Nagappa, Sharma, Basu, and Kishore Chopra. suggest a considerable hike of COVID infection among the patients with TB [30]. Also, Iyengar and Jain predicted that due to distributed attention during COVID-19 pandemic the tracking of TB patients and the vital activities for TB may be affected [23]. Besides, the work of Cilloni and Fu suggested to act aptly towards combating TB to avoid the adverse situation as a result of COVID-19 pandemic [18].

The integer-order derivative is local in nature, which means that the integer-order derivative is useful in understanding nature of the function at one point only and in the neighborhood of that point. The fractional-order derivatives can overcome the challenges imposed by traditional integer-order derivatives, and it also covers the aspects of integer order derivatives. There are various fractional order derivatives, the most notable of which are the Riemann–Liouville fractional derivative and the Caputo fractional derivative in applications. The Caputo fractional derivative is more suitable than the Riemann–Liouville fractional derivative as it requires initial conditions containing limit values at $t = 0$. The former gives more accurate and precise information regarding the complexities of numerous diseases. Fractional order models are better than conventional integer-order models because of hereditary properties and memory description [42, 43]. However, in reality, any phenomena of nature can be expressed better by a biological system of noninteger-order rather than integer-order models [17]. The fractional-order derivatives can apprehend nonlocal relationships in time and space, and they also offer more degree of freedom and a precise representation of nonlinear phenomena [17]. Saeed Ahmad et al. stated the use of the fusion method to develop a series solution for the analytical solution differential equations of fractional order which includes nonsingular derivative [8]. For TB disease, a three-strained noninteger order model is studied in [41]. A fractional order model is also used by [16] to analyze the dynamics of TB. Weronika used Lypanove theory to check whether a noninteger order system for tuberculosis is globally stable or not [50]. Moreover, [44] modeled a fraction order TB model using Caputo derivative from the factual data of Khyber Pakhtunkhwa.

This research paper establishes a fractional-order mathematical model in the Caputo sense to analyze the dynamical behavior of the spread of tuberculosis infection. The study represents various compartments of TB transmission infection for the dormant individuals (not diagnosed as infected) and individuals identified as infected with TB. The following system considers more intermediate compartments as diagnosis, high infection, and cure, to study different treatment and recovery aspects. Basic definitions are given in Sect. 2. Section 3 contains model formulation. The basic reproduction number and endemic equilibria are given in Sect. 4 and Sect. 5. In Sect. 6, parameter estimation and model fitting with the real data of the reported cases in India during the year 2000 to 2020 are given. The existence and uniqueness of the solution for the model is proved in Sect. 7. The numerical results of the approximate solution for the fractional-order model in the Caputo sense are given in Sect. 8. Numerical simulations with different scenarios for the model are given in Sect. 9, which are useful for the Ministry of Health and Family Welfare, Government of India to implement an action plan to control TB infection in India. Finally, conclusion is provided in Sect. 10.

## Prerequisites

This section discusses the prerequisites and elementary notions used throughout this paper.

### Definition 2.1

([35, 36])

For an integrable function f, the Caputo derivative of fractional order $\alpha \in (0,1)$ is given by

$$ {}^{C}D^{\alpha } f(t) = \frac{1}{\Gamma (m - \alpha )} \int _{0}^{t} \frac{f^{(m)}(v)}{(t - v)^{\alpha - m + 1}}\,dv,\quad m = [ \alpha ] + 1. $$

Also, the fractional integral of order *α* with $\operatorname{Re} (\alpha ) > 0$ is given by

$$ {}^{C}I^{\alpha } f(t) = \frac{1}{\Gamma (\alpha )} \int _{0}^{t} (t - v)^{\alpha - 1}f(v)\,dv. $$

### Definition 2.2

([11, 15])

For $f \in H^{1}(c,d)$ and $d > c$, the C-F derivative of fractional order $\alpha \in (0,1)$ for *f* is given by

$$ {}^{\mathrm{CF}}D^{\alpha } f(t) = \frac{M(\alpha )}{(1 - \alpha )} \int _{c}^{t} \exp \biggl( \frac{ - \alpha }{1 - \alpha } (t - v) \biggr)f'(v)\,dv, $$

where $t \ge 0$, $M(\alpha )$ is a normalization function that depends on *α* and $M(0) = M(1) = 1$. If $f \notin H^{1}(c,d)$ and $0 < \alpha < 1$, this derivative for $f \in L^{1}( - \infty ,d)$ is given by

$$ {}^{\mathrm{CF}}D^{\alpha } f(t) = \frac{\alpha M(\alpha )}{(1 - \alpha )} \int _{ - \infty }^{d} \bigl(f(t) - f(v)\bigr)\exp \biggl( \frac{ - \alpha }{1 - \alpha } (t - v) \biggr)f'(v)\,dv. $$

Also, the C-F fractional integral is presented by

$$ {}^{\mathrm{CF}}I^{\alpha } f(t) = \frac{2(1 - \alpha )}{(2 - \alpha )M(\alpha )}f(t) + \frac{2\alpha }{(2 - \alpha )M(\alpha )} \int _{0}^{t} f(v)\,dv. $$

### Definition 2.3

([35, 36])

The L. T. of Caputo fractional differential operator of order *α* is given by

$$ L \bigl[ {}^{C}D^{\alpha } f(t) \bigr](s) = s^{\alpha } Lf(t) - \sum_{i = 0}^{m - 1} s^{\alpha - i - 1}f^{(i)}(0), \quad m - 1 < \alpha \le m \in N, $$

which can also be written as

$$ L \bigl[ {}^{C}D^{\alpha } f(t) \bigr](s) = \frac{s^{m}L[f(t)] - s^{m - 1}f(0) - \cdots - f^{(m - 1)}}{s^{m - \alpha }}. $$

## Model formulation

**Figure 1:**
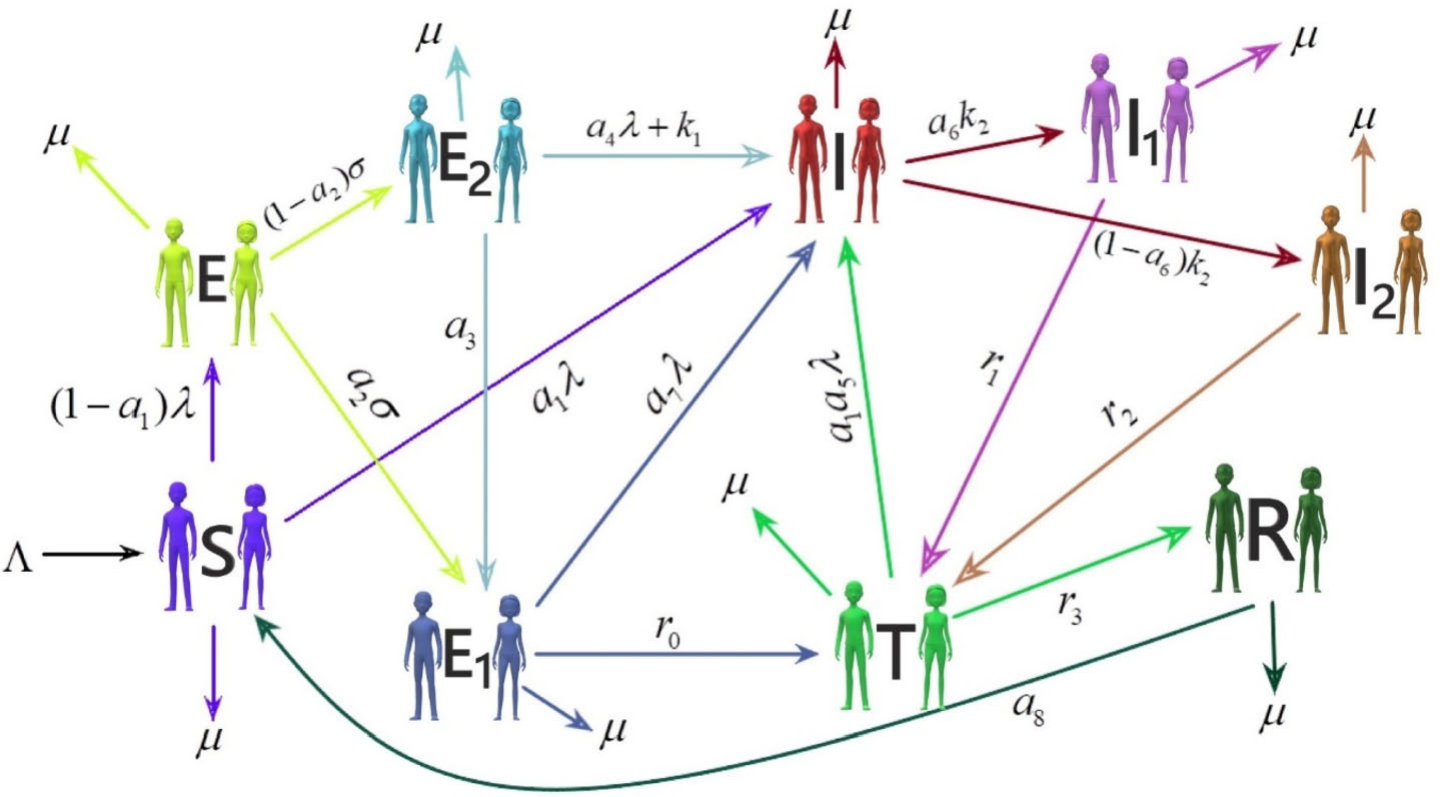
Transmission dynamics of TB

The compartmental model for the transmission dynamics of TB is represented graphically in Fig. 1. The list of state variables and their description with values are given in Table 1.

**Table 1:** List and description of the model parameters

Symbol	Description	Value	Reference
*λ*	Contact rate	0.1938	Fitted
Λ	Birth rate	2,00,00,063.55072464	Estimated [5]
*μ*	Natural mortality rate	1/69 = 0.014493	Estimated [4]
$a_{{1}}$	Rate at which *S*(*t*) is determined as *I*(*t*) and remaining people join *E*(*t*) compartment	0.7971	Fitted
$a_{{2}}$	Rate at which *E*(*t*) join $E_{1}(t)$ or $E_{2}(t)$ compartment	0.4131	Fitted
*σ*	Rate at which *E*(*t*) is classified as $E_{1}(t)$ or $E_{2}(t)$	0.1995	Fitted
$a_{{3}}$	Rate at which $E_{2}(t)$ join $E_{1}(t)$ compartment	0.3012	Fitted
$a_{{4}}$	Rate at which $E_{2}(t)$ join *I*(*t*) compartment	0.3410	Fitted
$k_{{1}}$	Progression rate at which $E_{2}(t)$ directly join *I*(*t*) compartment	0.3269	Fitted
$a_{{5}}$	Rate at which *T*(*t*) join *I*(*t*) compartment	0.2848	Fitted
$a_{{6}}$	Rate at which *I*(*t*) join $I_{1}(t)$ or $I_{2}(t)$ compartment	0.6102	Fitted
${k}_{{2}}$	Rate at which *I*(*t*) is classified as $I_{1}(t)$ or $I_{2}(t)$ compartment	0.4213	Fitted
$a_{{7}}$	Rate at which $E_{1}(t)$ join *I*(*t*) compartment	0.5654	Fitted
$a_{{8}}$	Rate at which *R*(*t*) becomes *S*(*t*) individual	0.7139	Fitted
$r_{{0}}$	Rate at which $E_{1}(t)$ join *T*(*t*) compartment	0.1512	Fitted
$r_{{1}}$	Rate at which $I_{1}(t)$ join *T*(*t*) compartment	0.2177	Fitted
$r_{{2}}$	Rate at which $I_{2}(t)$ join *T*(*t*) compartment	0.4913	Fitted
$r_{{3}}$	Rate at which *T*(*t*) becomes *R*(*t*) individual	0.3761	Fitted

The population is separated into nine compartments which are classified based on the infection status. Here, *λ* is the contact rate. At time *t*, $S(t)$ represents the number of susceptible individuals at risk of getting infected. $a_{1}\lambda $ is the rate of the susceptible individual classified as infected and joining $I(t)$ class. $E(t)$ is the number of the new latently infected individual being in sufficient contact with an infected person but not contagious. $a_{2}\sigma $ is the rate at which the new latently infected individual joins the diagnosed latently infected $E_{1}(t)$ and others associated with the undiagnosed latently infected $E_{2}(t)$ class based on the TB test result. The undiagnosed actively infected people $I(t)$ are those who become contagious and can spread infection to others. $a_{7}\lambda $ and $a_{4}\lambda + k_{1}$ are the rates at which individuals from $E_{1}(t)$ and $E_{2}(t)$ become actively infected. The individual from $I(t)$ class diagnosed as actively infected with rate $a_{6}k_{2}$ joins the class $I_{1}(t)$, which represents the individuals diagnosed as actively infected with speedy treatment. The people diagnosed as actively infected but delayed in treatment are joining $I_{2}(t)$ at rate $(1 - a_{6})k_{2}$. $r_{0}$, $r_{1}$, and $r_{2}$ are the rates at which infected individuals from $E_{1}(t)$, $I_{1}(t)$, and $I_{2}(t)$ started treatment and moved to treatment compartment $T(t)$. $R(t)$ represents the number of recovered or removed individuals as well as those who stopped treatments after partial recovery. A person who has completed treatment and recovered moves to $R(t)$ at rate $r_{3}$. In MDR and XDR compartments of TB, an individual from $T(t)$ class is moved to $I(t)$ compartment at rate $a_{1}a_{5}\lambda $. The recovered people can become susceptible at the rate $a_{8}$. The integer-order model is unable to represent the dynamics of the real-world problem between two points. The fractional-order mathematical model is a more reliable and powerful tool for minimizing error created by neglected parameters in modeling [27, 34]. The fractional order dynamical system of the TB infection model using Caputo derivative of order $\alpha \in (0,1)$ is given by

1 $$\begin{aligned}& {}^{C}D_{t}^{\alpha } S = \Lambda + a_{8}R - (\lambda + \mu )S, \\& {}^{C}D_{t}^{\alpha } E = (1 - a_{1})\lambda (S + a_{5}T) - (\sigma + \mu )E, \\& {}^{C}D_{t}^{\alpha } E_{1} = a_{2} \sigma E + a_{3}E_{2} - a_{7}\lambda E_{1} - (r_{0} + \mu )E_{1}, \\& {}^{C}D_{t}^{\alpha } E_{2} = (1 - a_{2})\sigma E - (a_{4}\lambda + k_{1} + a_{3} + \mu )E_{2} , \\& {}^{C}D_{t}^{\alpha } I = a_{1}\lambda (S + a_{5}T) + a_{7}\lambda E_{1} + (a_{4} \lambda + k_{1})E_{2} - (k_{2} + \mu )I , \\& {}^{C}D_{t}^{\alpha } I_{1} = a_{6}k_{2}I - (r_{1} + \mu )I_{1}, \\& {}^{C}D_{t}^{\alpha } I_{2} = (1 - a_{6})k_{2}I - (r_{2} + \mu )I_{2}, \\& {}^{C}D_{t}^{\alpha } T = r_{0}E_{1} + r_{1}I_{1} + r_{2}I_{2} - (a_{1}a_{5}\lambda + r_{3} + \mu )T, \\& {}^{C}D_{t}^{\alpha } R = r_{3}T - (a_{8} + \mu )R \end{aligned}$$

with the nonnegative initial condition

2 $$\begin{aligned}& S(0) \ge 0,\qquad E(0) \ge 0,\qquad E_{1}(0) \ge 0,\qquad E_{2}(0) \ge 0, \\& I(0) \ge 0,\qquad I_{1}(0) \ge 0,\qquad I_{2}(0) \ge 0, \\& T(0) \ge 0,\qquad R(0) \ge 0. \end{aligned}$$

Therefore, we can observe that the solution of system (1) is nonnegative and bounded [29].

The overall dynamics of the population attained by adding all six equations of model (1) is as follows:

$$ \frac{dN}{dt} = \Lambda - \mu N. $$

System (1) is moderated by substituting the Caputo fractional time-derivative. In this moderate system, the dimension of the system will not remain the same for the right and left sides. We use Ψ- an auxiliary parameter to resolve this problem. As per the discussion, the fractional-order model for $t > 0$ and $\alpha \in (0,1)$ with the same initial conditions is given by

3 $$\begin{aligned}& \Psi ^{\alpha - 1}{}^{C}D_{t}^{\alpha } S(t) = \Lambda + a_{8}R - (\lambda + \mu )S, \\& \Psi ^{\alpha - 1}{}^{C}D_{t}^{\alpha } E(t) = (1 - a_{1})\lambda (S + a_{5}T) - (\sigma + \mu )E , \\& \Psi ^{\alpha - 1}{}^{C}D_{t}^{\alpha } E_{1}(t) = a_{2}\sigma E + a_{3}E_{2} - a_{7}\lambda E_{1} - (r_{0} + \mu )E_{1}, \\& \Psi ^{\alpha - 1}{}^{C}D_{t}^{\alpha } E_{2}(t) = (1 - a_{2})\sigma E - (a_{4}\lambda + k_{1} + a_{3} + \mu )E_{2}, \\& \Psi ^{\alpha - 1}{}^{C}D_{t}^{\alpha } I(t) = a_{1}\lambda (S + a_{5}T) + a_{7}\lambda E_{1} + (a_{4}\lambda + k_{1})E_{2} - (k_{2} + \mu )I , \\& \Psi ^{\alpha - 1}{}^{C}D_{t}^{\alpha } I_{1} = a_{6}k_{2}I - (r_{1} + \mu )I_{1}, \\& \Psi ^{\alpha - 1}{}^{C}D_{t}^{\alpha } I_{2} = (1 - a_{6})k_{2}I - (r_{2} + \mu )I_{2}, \\& \Psi ^{\alpha - 1}{}^{C}D_{t}^{\alpha } T = r_{0}E_{1} + r_{1}I_{1} + r_{2}I_{2} - (a_{1}a_{5}\lambda + r_{3} + \mu )T, \\& \Psi ^{\alpha - 1}{}^{C}D_{t}^{\alpha } R = r_{3}T - (a_{8} + \mu )R. \end{aligned}$$

The feasible region for model (1) is given by

4 $$ \Omega = \biggl\{ S(t),E(t),E_{1}(t),E_{2}(t),I(t),I_{1}(t),I_{2}(t),T(t),R(t) \in \mathbb{R}_{ +}^{9}:N(t) \le \frac{\Lambda }{\mu } \biggr\} . $$

We prove that the closed set Ω is the feasible region of system (3).

### Lemma 3.1

*The closed set* Ω *is a positive invariant concerning fractional system* (3).

### Proof

We add all the terms in system (3) to obtain the overall population in the fractional order, i.e., $\Psi ^{\alpha - 1}{}^{C}D_{t}^{\alpha } N(t) = \Lambda - \mu N(t)$, where $N(t) = S(t) + E(t) + E_{1}(t) + E_{2}(t) + I(t) + I_{1}(t) + I_{2}(t) + T(t) + R(t)$.

To obtain the population size, we use the Laplace transform as follows:

$$ N(t) = N(0)E_{\alpha } \bigl( - \mu \Psi ^{1 - \alpha } t^{\alpha } \bigr) + \int _{0}^{t} \Lambda \Psi ^{1 - \alpha } \theta ^{\alpha - 1}E_{\alpha ,\alpha } \bigl( - \mu \Psi ^{1 - \alpha } \theta ^{\alpha } \bigr)\,d\theta , $$

where $N(0)$ is the initial population size. After simplifying we get

$$\begin{aligned} N(t)& = N(0)E_{\alpha } \bigl( - \mu \Psi ^{1 - \alpha } t^{\alpha } \bigr) + \int _{0}^{t} \Lambda \Psi ^{1 - \alpha } \theta ^{\alpha - 1}\sum_{i = 0}^{\infty } \frac{( - 1)^{i}\mu ^{i}\Psi ^{i(1 - \alpha )}\theta ^{i\alpha }}{\Gamma (i\alpha + \alpha )}\,d\theta \\ &= \frac{\Lambda }{\mu } + E_{\alpha } \bigl( - \mu \Psi ^{1 - \alpha } t^{\alpha } \bigr) \biggl( N(0) - \frac{\Lambda }{\mu } \biggr). \end{aligned}$$

Thus, if $N(0) \le \frac{\Lambda }{\mu } $, then for $t > 0$, $N(t) \le \frac{\Lambda }{\mu } $.

The following table represents the parametric values used for model (1) fitted or estimated using data available and reported cases of TB infection in India.

Consequently, the closed set Ω is positive invariant concerning fractional-order model (3). The solutions of system (1) are known as equilibrium points, and they have two equilibrium points as follows:

1. Infection-free equilibrium points;
2. Endemic equilibrium points,

which are discussed in the following sections. □

## Infection-free equilibrium points

The basic reproduction number $\Re _{0}$ (also known as threshold parameter) is used to analyze the average number of secondary infected users rising from an average primary infected users in entirely susceptible population for the stability of system (1). $\Re _{0}$ is obtained by using the next generation matrix [47]. The new infectious rates represented using matrix *F*, and other transferred rates within compartments by matrix *V* are given as follows:

$$\begin{array}{c} F=\left[\begin{array}{ccccccc} 0 & 0 & 0 & 0 & 0 & 0 & \lambda a_{5}\\ 0 & 0 & 0 & 0 & 0 & 0 & 0\\ 0 & 0 & 0 & 0 & 0 & 0 & 0\\ 0 & 0 & 0 & 0 & 0 & 0 & 0\\ 0 & 0 & 0 & 0 & 0 & 0 & 0\\ 0 & 0 & 0 & 0 & 0 & 0 & 0\\ 0 & 0 & 0 & 0 & 0 & 0 & 0 \end{array}\right],\\ V=\left(\begin{array}{ccccccc} \sigma +\mu & 0 & 0 & 0 & 0 & 0 & 0\\ -a_{2}\sigma & a_{7}\lambda +r_{0}+\mu & -a_{3} & 0 & 0 & 0 & 0\\ -\sigma (1-a_{2}) & 0 & a_{4}\lambda +k_{1}+a_{3}+\mu & 0 & 0 & 0 & 0\\ 0 & -a_{7}\lambda & -(a_{4}\lambda +k_{1}) & k_{2}+\mu & 0 & 0 & 0\\ 0 & 0 & 0 & -a_{6}k_{2} & r_{1}+\mu & 0 & 0\\ 0 & 0 & 0 & -k_{2}(1-a_{6}) & 0 & r_{2}+\mu & 0\\ 0 & -r_{0} & 0 & 0 & -r_{1} & -r_{2} & a_{1}a_{5}\lambda +r_{3}+\mu \end{array}\right). \end{array}$$

The basic reproduction number of system (3) is given by the spectral radius

$$\begin{array}{rl} \gamma \left(FV^{-1}\right) & ={ℜ}_{0}\\ & =\frac{\left(\begin{array}{c} \lambda a_{5}(k_{2}+\mu )(r_{2}+\mu )(a_{3}r_{0}\sigma (1-a_{2})+r_{0}a_{2}\sigma (a_{4}\lambda +k_{1}+a_{3}+\mu ))\\ +\lambda a_{5}k_{2}(r_{1}a_{6}(r_{2}+\mu )\\ +r_{2}(1-a_{6}))(\sigma (1-a_{2}))((a_{4}\lambda +\mu )(a_{7}\lambda +r_{0}+\mu )+a_{3}a_{7}\lambda )\\ +(a_{2}a_{7}\lambda \sigma )(a_{4}\lambda +k_{1}+a_{3}+\mu ) \end{array}\right)}{\left(k_{2}+\mu )(r_{2}+\mu )(\sigma +\mu )(a_{7}\lambda +r_{0}+\mu )(a_{4}\lambda +k_{1}+a_{3}+\mu )(a_{1}a_{5}\lambda +r_{3}+\mu \right)}. \end{array}$$

The basic reproduction number is obtained as $\Re _{0} = 1.7307$ using the parameter values given in Table 1 for India.

If $\Re _{0} > 1$ then the disease will persist in the community since one diseased individual will infect more than one susceptible individual on average. This is possible if

$$ (k_{2} + \mu ) (r_{2} + \mu ) (\sigma + \mu ) (a_{7}\lambda + r_{0} + \mu ) (a_{4}\lambda + k_{1} + a_{3} + \mu ) (a_{1}a_{5} \lambda + r_{3} + \mu ) < 1, $$

i.e., the values of parameters $k_{1}$, $k_{2}$, $a_{1}$, $a_{4}$, $a_{5}$,and $a_{7}$ decrease, which means that the latently or actively infected individual remains in the population and may spread the infection to others. Also, the decrement of the parameters $r_{2}$, $r_{3}$, *σ*, $r_{0}$, and $a_{3}$ means that the infected individuals and relapse cases are not taking proper treatment or left treatments before recovery and may propagate infection to others.

If $\Re _{0} < 1$ then on average one diseased individual can only infect one other person, and the disease will eventually die out.

### Theorem 4.1

*The TB*-*infection free equilibrium* $E_{0} = ( \frac{\Lambda }{\mu },0,0,0,0,0,0,0,0 )$ *for model* (3) *is locally asymptotically instable if* $\Re _{0} > 1$.

### Proof

To obtain the TB-infection free equilibrium at a point $E_{0}$, the Jacobian matrix [19, 22] is as follows:

$$J=\left(\begin{array}{ccccccccc} -(\lambda +\mu ) & 0 & 0 & 0 & 0 & 0 & 0 & 0 & a_{8}\\ (1-a_{1})\lambda & -(\sigma +\mu ) & 0 & 0 & 0 & 0 & 0 & (1-a_{1})a_{5}\lambda & 0\\ 0 & a_{2}\sigma & -(a_{7}\lambda +r_{0}+\mu ) & a_{3} & 0 & 0 & 0 & 0 & 0\\ 0 & (1-a_{2})\sigma & 0 & -(a_{4}\lambda +k_{1}+a_{3}+\mu ) & 0 & 0 & 0 & 0 & 0\\ a_{1}\lambda & 0 & a_{7}\lambda & \left(a_{4}\lambda +k_{1}\right) & -(k_{2}+\mu ) & 0 & 0 & a_{1}a_{5}\lambda & 0\\ 0 & 0 & 0 & 0 & a_{6}k_{2} & -(r_{1}+\mu ) & 0 & 0 & 0\\ 0 & 0 & 0 & 0 & (1-a_{6})k_{2} & 0 & -(r_{2}+\mu ) & 0 & 0\\ 0 & 0 & r_{0} & 0 & 0 & r_{1} & r_{2} & -(a_{1}a_{5}\lambda +r_{3}+\mu ) & 0\\ 0 & 0 & 0 & 0 & 0 & 0 & 0 & r_{3} & -(a_{8}+\mu ) \end{array}\right).$$

Define $s(J)=\max\{\Re\lambda : \lambda \text{ is an eigenvalue of }J\}$, where $s(J)$ is the simple eigenvalue of matrix *J* with positive eigenvector, then we have $\Re _{0} < 1 \Leftrightarrow s(J) < 0$, see [47] for more details. Therefore,

$$\begin{aligned} s(J) ={}& \max \bigl\{ - (\lambda + \mu ), - (\sigma + \mu ), - (a_{7}\lambda + r_{0} + \mu ), \\ &{}- (a_{4}\lambda + k_{1} + a_{3} + \mu ), - (k_{2} + \mu ), - (r_{1} + \mu ), \\ &{}- (r_{2} + \mu ), - (a_{1}a_{5}\lambda + r_{3} + \mu ), - (a_{8} + \mu )\bigr\} \\ < {}& 0. \end{aligned}$$

Hence, if $\Re _{0} < 1$, then $s(J) < 0$, $E_{0}$ is locally asymptotically instable. □

## Endemic equilibria

We use the following equations to determine the equilibrium points for the fractional-order model (3):

$$ \begin{aligned} {}^{C}D^{\alpha } S(t) &= {}^{C}D^{\alpha } E(t) = {}^{C}D^{\alpha } E_{1}(t) = {}^{C}D^{\alpha } E_{2}(t) = {}^{C}D^{\alpha } I(t) = {}^{C}D^{\alpha } I_{1}(t) = {}^{C}D^{\alpha } I_{2}(t) \\ &= {}^{C}D^{\alpha } T(t) = {}^{C}D^{\alpha } R(t) = 0. \end{aligned} $$

The algebraic solution of the equation provides equilibrium points of the system, and if $\Re _{0} > 1$, then system (3) has a positive endemic equilibrium $E_{1}^{*} = (S^{*},E^{*},E_{1}^{*},E_{2}^{*},I^{*},I_{1}^{*},I_{2}^{*},T^{*}, R^{*})$ and the Jacobian is given as

$$J_{1}=\left(\begin{array}{ccccccccc} -(\lambda +\mu ) & 0 & 0 & 0 & 0 & 0 & 0 & -\lambda a_{5} & a_{8}\\ (1-a_{1})\lambda & -(\sigma +\mu ) & 0 & 0 & 0 & 0 & 0 & (1-a_{1})a_{5}\lambda & 0\\ 0 & a_{2}\sigma & -(a_{7}\lambda +r_{0}+\mu ) & a_{3} & 0 & 0 & 0 & 0 & 0\\ 0 & (1-a_{2})\sigma & 0 & -(a_{4}\lambda +k_{1}+a_{3}+\mu ) & 0 & 0 & 0 & 0 & 0\\ a_{1}\lambda & 0 & a_{7}\lambda & \left(a_{4}\lambda +k_{1}\right) & -(k_{2}+\mu ) & 0 & 0 & a_{1}a_{5}\lambda & 0\\ 0 & 0 & 0 & 0 & a_{6}k_{2} & -(r_{1}+\mu ) & 0 & 0 & 0\\ 0 & 0 & 0 & 0 & (1-a_{6})k_{2} & 0 & -(r_{2}+\mu ) & 0 & 0\\ 0 & 0 & r_{0} & 0 & 0 & r_{1} & r_{2} & -(a_{1}a_{5}\lambda +r_{3}+\mu ) & 0\\ 0 & 0 & 0 & 0 & 0 & 0 & 0 & r_{3} & -(a_{8}+\mu ) \end{array}\right),$$

where $S* = \frac{(\Lambda + a_{8}R*)}{(\lambda + \mu )}$, $E* = \frac{\lambda (1 - a_{1})(S* + a_{5}T*)}{(\sigma + \mu )}$, $E_{1}* = \frac{1}{(a_{7}\lambda + r_{0} + \mu )}(a_{2}\sigma E* + a_{3}E_{2}*)$,

$$\begin{aligned}& E_{2}* = \frac{\sigma (1 - a_{2})}{(a_{4}\lambda + k_{1} + a_{3} + \mu )}E*, \\& I* = \frac{1}{(k_{2} + \mu )} \bigl( a_{1}\lambda (S* + a_{5}T*) + a_{7}\lambda E_{1}* + (a_{4}\lambda + k_{1})E_{2}* \bigr), \\& I_{1}* = \biggl( \frac{a_{6}k_{2}}{r_{1} + \mu } \biggr)I*, \qquad I_{2}* = \biggl( \frac{(1 - a_{6})k_{2}}{r_{2} + \mu } \biggr)I*, \\& T* = \frac{(a_{8} + \mu )}{r_{3}} \times R*\quad \text{or} \quad \frac{ ( r_{0}E_{1}* + r_{1}I_{1}* + r_{2}I_{2}* )}{ ( a_{1}a_{5}\lambda + r_{3} + \mu )} \\& R* = ((r_{0}\Lambda )\bigl\{ \bigl(a_{2}(a_{4} \lambda + k_{1} + \mu ) + a_{3}\bigr) \bigl(r_{0} \lambda \sigma (r_{2} + \mu ) \bigl(k_{2} \bigl(r_{1}a_{6} + (1 - a_{6})r_{2} \bigr) (a_{7}\lambda + r_{0} + \mu )\bigr) \\& \hphantom{R* =}{}\times \bigl((1 - a_{2}) (a_{4}\lambda + k_{1} + a_{3} + \mu )\bigr)\bigr\} \times (\Re _{0} - 1)\bigr) \\& \hphantom{R* =}{}/\bigl(\bigl(r_{1}a_{6}(r_{2} + \mu ) (\sigma + \mu ) (a_{7}\lambda + r_{0} + \mu ) - \lambda \sigma (a_{4}\lambda + k_{1} + a_{3} + \mu ) (1 - a_{1}) (1 - a_{2})\bigr) \\& \hphantom{R* =}{}\times \Re _{0}\bigr). \end{aligned}$$

From the above equations, we observe that the endemic equilibrium $E_{1}^{*} = (S^{*},E^{*},E_{1}^{*},E_{2}^{*}, I^{*},I_{1}^{*},I_{2}^{*},T^{*},R^{*})$ only exists if $\Re _{0} > 0$.

## Model fitting and parameter estimation

**Figure 2:**
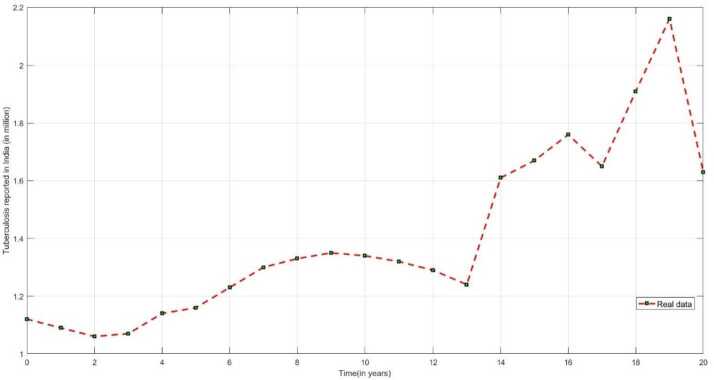
The number of TB infected individuals reported in India from Central Tuberculosis Division, Government of India under National Tuberculosis Elimination Programme

Figure 2 represents the reported cases of TB infected people in India from the year 2000 to 2020 [1, 3] by the Central Tuberculosis Division, Government of India under National Tuberculosis Elimination Programme.

**Figure 3:**
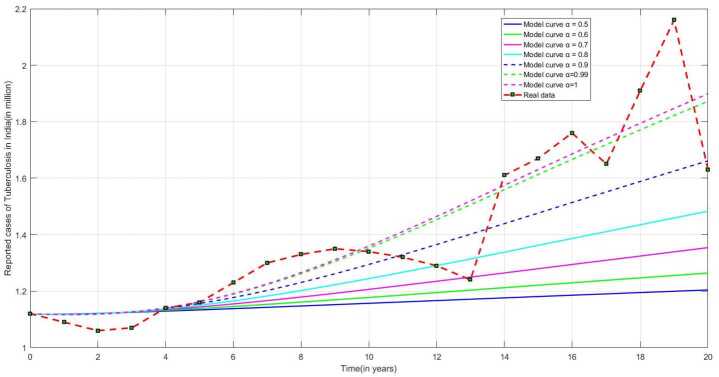
Real data fitting using model (1) for $\alpha = 0.5, 0.6, 0.7, 0.8, 0.9, 0.99, 1$

In order to obtain a good fit to the real data, we use the least square curve fitting algorithm given by Khan et al. [26] for our model except the birth rate Λ and the natural mortality rate *μ*. The average life span in India is 69 years mentioned in Press Information Bureau, Government of India [4]. The natural mortality rate is considered as the reciprocal of average life expectancy of the people of India and estimated value as $\mu = 1/69$ per year. To estimate the birth rate, we considered the population of India as $\Lambda /\mu =1{,}380{,}004{,}385$ for the year 2020 [5]. So, the limiting population in absence of infection is obtained as $\Lambda =20{,}000{,}063.55072464$ per year. Figure 3 represent the model fitting to the real data for various fractional order.

**Figure 4:**
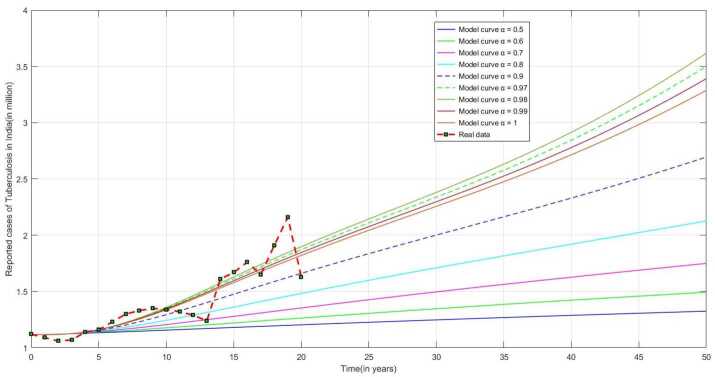
Predicted behavior of TB infection for long period of time in India for our model (1)

From Fig. 4, we can see the long-term trend in the number of reported cases of tuberculosis infection in India, which demonstrates a significant increase in the number of recorded cases and gives a terrible sign to the Ministry of Health and Family Welfare, Government of India. The model parameters given in Table 1 are calculated from the reported data and will be employed in numerical simulations.

## Existence and uniqueness of solution

In this section, we use the generalized Adams–Bashforth–Moulton technique given in [35] to prove the uniqueness of the solution for system (3) as follows:

$$\begin{aligned}& \Psi ^{\alpha - 1}{}^{C}D_{t}^{\alpha } S(t) = Q_{1}\bigl(t,S(t)\bigr), \\& \Psi ^{\alpha - 1}{}^{C}D_{t}^{\alpha } E(t) = Q_{2}\bigl(t,E(t)\bigr), \\& \Psi ^{\alpha - 1}{}^{C}D_{t}^{\alpha } E_{1}(t) = Q_{3}\bigl(t,E_{1}(t)\bigr), \\& \Psi ^{\alpha - 1}{}^{C}D_{t}^{\alpha } E_{2}(t) = Q_{4}\bigl(t,E_{2}(t)\bigr), \\& \Psi ^{\alpha - 1}{}^{C}D_{t}^{\alpha } I(t) = Q_{5}\bigl(t,I(t)\bigr), \\& \Psi ^{\alpha - 1}{}^{C}D_{t}^{\alpha } I_{1}(t) = Q_{6}\bigl(t,I_{1}(t)\bigr), \\& \Psi ^{\alpha - 1}{}^{C}D_{t}^{\alpha } I_{2}(t) = Q_{7}\bigl(t,I_{2}(t)\bigr), \\& \Psi ^{\alpha - 1}{}^{C}D_{t}^{\alpha } T(t) = Q_{8}\bigl(t,T(t)\bigr) , \\& \Psi ^{\alpha - 1}{}^{C}D_{t}^{\alpha } R(t) = Q_{9}\bigl(t,R(t)\bigr). \end{aligned}$$

By using Lemma 3.1, the system is given as

5 $$\begin{aligned}& S(t) - S(0) = \frac{\Psi ^{1 - \alpha }}{\Gamma \alpha } \int _{0}^{t} Q_{1}(\tau ,S) (t - \tau )^{\alpha - 1}\,d\tau, \\& E(t) - E(0) = \frac{\Psi ^{1 - \alpha }}{\Gamma \alpha } \int _{0}^{t} Q_{2}(\tau ,E) (t - \tau )^{\alpha - 1}\,d\tau , \\& E_{1}(t) - E_{1}(0) = \frac{\Psi ^{1 - \alpha }}{\Gamma \alpha } \int _{0}^{t} Q_{3}(\tau ,E_{1}) (t - \tau )^{\alpha - 1}\,d\tau , \\& E_{2}(t) - E_{2}(0) = \frac{\Psi ^{1 - \alpha }}{\Gamma \alpha } \int _{0}^{t} Q_{4}(\tau ,E_{2}) (t - \tau )^{\alpha - 1}\,d\tau , \\& I(t) - I(0) = \frac{\Psi ^{1 - \alpha }}{\Gamma \alpha } \int _{0}^{t} Q_{5}(\tau ,I) (t - \tau )^{\alpha - 1}\,d\tau , \\& I_{1}(t) - I_{1}(0) = \frac{\Psi ^{1 - \alpha }}{\Gamma \alpha } \int _{0}^{t} Q_{6}(\tau ,I_{1}) (t - \tau )^{\alpha - 1}\,d\tau , \\& I_{2}(t) - I_{2}(0) = \frac{\Psi ^{1 - \alpha }}{\Gamma \alpha } \int _{0}^{t} Q_{7}(\tau ,I_{2}) (t - \tau )^{\alpha - 1}\,d\tau , \\& T(t) - T(0) = \frac{\Psi ^{1 - \alpha }}{\Gamma \alpha } \int _{0}^{t} Q_{8}(\tau ,I) (t - \tau )^{\alpha - 1}\,d\tau, \\& R(t) - R(0) = \frac{\Psi ^{1 - \alpha }}{\Gamma \alpha } \int _{0}^{t} Q_{9}(\tau ,R) (t - \tau )^{\alpha - 1}\,d\tau . \end{aligned}$$

In the ensuing theorem the kernels $Q_{i}$, $i = 1,2,3,4,5,6,7,8,9$, satisfy the Lipschitz condition and contraction.

### Theorem 7.1

*The kernel* $Q_{1}$ *satisfies the Lipschitz condition and contraction if the inequality given below holds* $0 \le \lambda + \mu < 1$.

### Proof

For *S* and $S_{*}$ we have

$$ \bigl\Vert Q_{1}(t,S) - Q_{1}(t,S_{*}) \bigr\Vert \le (\lambda + \mu ) \Vert S - S_{*} \Vert . $$

Suppose that $d_{1} = \lambda + \mu $, where $\|S\| \le M_{1}$, $\|E\| \le M_{2}$, $\|E_{1}\| \le M_{3}$, $\|E_{2}\| \le M_{4}$, $\|I\| \le M_{5}$, $\|I_{1}\| \le M_{6}$, $\|I_{2}\| \le M_{7} \|T\| \le M_{8}$ and $\|R\| \le M_{9}$ is a bounded function. So

6 $$ \bigl\Vert Q_{1}(t,S) - Q_{1}(t,S_{*}) \bigr\Vert \le d_{1} \bigl\Vert S(t) - S_{*}(t) \bigr\Vert . $$

Thus, for $Q_{1}$ the Lipchitz condition is obtained, and if $0 \le \lambda + \mu < 1$ then $Q_{1}$ is a contraction.

Similarly, the Lipschitz condition for $Q_{i}$, $i = 2,3,4,5,6,7,8,9$, is given as follows:

$$\begin{aligned}& \bigl\Vert Q_{2}(t,E) - Q_{2}(t,E_{*}) \bigr\Vert \le d_{2} \bigl\Vert E(t) - E_{*}(t) \bigr\Vert , \\& \bigl\Vert Q_{3}(t,E_{1}) - Q_{3}(t,E_{1*}) \bigr\Vert \le d_{3} \bigl\Vert E_{1}(t) - E_{1*}(t) \bigr\Vert , \\& \bigl\Vert Q_{4}(t,E_{2}) - Q_{4}(t,E_{2*}) \bigr\Vert \le d_{4} \bigl\Vert E_{2}(t) - E_{2*}(t) \bigr\Vert , \\& \bigl\Vert Q_{5}(t,I) - Q_{5}(t,I_{*}) \bigr\Vert \le d_{5} \bigl\Vert I(t) - I_{*}(t) \bigr\Vert , \\& \bigl\Vert Q_{6}(t,I_{1}) - Q_{6}(t,I_{1*}) \bigr\Vert \le d_{6} \bigl\Vert I_{1}(t) - I_{1*}(t) \bigr\Vert , \\& \bigl\Vert Q_{7}(t,I_{2}) - Q_{7}(t,I_{2*}) \bigr\Vert \le d_{7} \bigl\Vert I_{2}(t) - I_{2*}(t) \bigr\Vert , \\& \bigl\Vert Q_{8}(t,T) - Q_{8}(t,T_{*}) \bigr\Vert \le d_{8} \bigl\Vert T(t) - T_{*}(t) \bigr\Vert , \\& \bigl\Vert Q_{9}(t,R) - Q_{9}(t,R_{*}) \bigr\Vert \le d_{9} \bigl\Vert R(t) - R_{*}(t) \bigr\Vert , \end{aligned}$$

where $d_{2} = \sigma + \mu $, $d_{3} = a_{7}\lambda + r_{0} + \mu $, $d_{4} = a_{4}\lambda + k_{1} + a_{3} + \mu $, $d_{5} = k_{2} + \mu $, $d_{6} = r_{1} + \mu $, $d_{7} = r_{2} + \mu $, $d_{8} = a_{1}a_{5}\lambda + r_{3} + \mu $,and $d_{9} = a_{8} + \mu $ are bounded functions, if $0 \le d_{i} < 1$, $i = 2,3,4,5,6,7,8,9$, then $Q_{i}$, $i = 2,3,4,5,6,7,8,9$, are contractions. According to system (5), consider the following recursive forms:

$$\begin{aligned}& P_{1n}(t) = S_{n}(t) - S_{n - 1}(0) = \frac{\Psi ^{1 - \alpha }}{\Gamma \alpha } \int _{0}^{t} \bigl[Q_{1}(\tau ,S_{n - 1}) - Q_{1}(\tau ,S_{n - 2})\bigr](t - \tau )^{\alpha - 1}\,d\tau, \\& P_{2n}(t) = E_{n}(t) - E_{n - 1}(0) = \frac{\Psi ^{1 - \alpha }}{\Gamma \alpha } \int _{0}^{t} \bigl[Q_{2}(\tau ,E_{n - 1}) - Q_{2}(\tau ,E_{n - 2})\bigr](t - \tau )^{\alpha - 1}\,d\tau, \\& P_{3n}(t) = (E_{1})_{n}(t) - (E_{1})_{n - 1}(0) \\& \hphantom{P_{3n}(t)}= \frac{\Psi ^{1 - \alpha }}{\Gamma \alpha } \int _{0}^{t} \bigl[Q_{3}\bigl(\tau ,(E_{1})_{n - 1}\bigr) - Q_{3}\bigl(\tau ,(E_{1})_{n - 2}\bigr)\bigr](t - \tau )^{\alpha - 1}\,d\tau, \\& P_{4n}(t) = (E_{2})_{n}(t) - (E_{2})_{n - 1}(0) \\& \hphantom{P_{4n}(t)} = \frac{\Psi ^{1 - \alpha }}{\Gamma \alpha } \int _{0}^{t} \bigl[Q_{4}\bigl(\tau ,(E_{2})_{n - 1}\bigr) - Q_{4}\bigl(\tau ,(E_{2})_{n - 2}\bigr)\bigr](t - \tau )^{\alpha - 1}\,d\tau, \\& P_{5n}(t) = I_{n}(t) - I_{n - 1}(0) = \frac{\Psi ^{1 - \alpha }}{\Gamma \alpha } \int _{0}^{t} \bigl[Q_{5}(\tau ,I_{n - 1}) - Q_{5}(\tau ,I_{n - 2})\bigr](t - \tau )^{\alpha - 1}\,d\tau, \\& P_{6n}(t) = (I_{1})_{n}(t) - (I_{1})_{n - 1}(0) = \frac{\Psi ^{1 - \alpha }}{\Gamma \alpha } \int _{0}^{t} \bigl[Q_{6}\bigl(\tau ,(I_{1})_{n - 1}\bigr) - Q_{6}\bigl(\tau ,(I_{1})_{n - 2}\bigr)\bigr](t - \tau )^{\alpha - 1}\,d\tau, \\& P_{7n}(t) = (I_{2})_{n}(t) - (I_{2})_{n - 1}(0) = \frac{\Psi ^{1 - \alpha }}{\Gamma \alpha } \int _{0}^{t} \bigl[Q_{7}\bigl(\tau ,(I_{2})_{n - 1}\bigr) - Q_{7}\bigl(\tau ,(I_{2})_{n - 2}\bigr)\bigr](t - \tau )^{\alpha - 1}\,d\tau, \\& P_{8n}(t) = T_{n}(t) - T_{n - 1}(0) = \frac{\Psi ^{1 - \alpha }}{\Gamma \alpha } \int _{0}^{t} \bigl[Q_{8}(\tau ,T_{n - 1}) - Q_{8}(\tau ,T_{n - 2})\bigr](t - \tau )^{\alpha - 1}\,d\tau, \\& P_{9n}(t) = R_{n}(t) - R_{n - 1}(0) = \frac{\Psi ^{1 - \alpha }}{\Gamma \alpha } \int _{0}^{t} \bigl[Q_{9}(\tau ,R_{n - 1}) - Q_{9}(\tau ,R_{n - 2})\bigr](t - \tau )^{\alpha - 1}\,d\tau \end{aligned}$$

with the initial conditions

$$\begin{aligned}& S_{0}(t) = S(0),\qquad E_{0}(t) = E(0),\qquad (E_{1})_{0}(t) = (E_{1}) (0), \\& (E_{2})_{0}(t) = (E_{2}) (0),\qquad I_{0}(t) = I(0), \\& (I_{1})_{0}(t) = (I_{1}) (0),\qquad (I_{2})_{0}(t) = (I_{2}) (0),\qquad T_{0}(t) = T(0)\quad \text{and}\quad R_{0}(t) = R(0). \end{aligned}$$

We take the norm of the first equation in the above system, then

$$\begin{aligned} \bigl\Vert P_{1n}(t) \bigr\Vert =& \bigl\Vert S_{n}(t) - S_{n - 1}(0) \bigr\Vert \\ =& \biggl\Vert \frac{\Psi ^{1 - \alpha }}{\Gamma \alpha } \int _{0}^{t} \bigl[Q_{1}(\tau ,S_{n - 1}) - Q_{1}(\tau ,S_{n - 2})\bigr](t - \tau )^{\alpha - 1}\,d\tau \biggr\Vert \\ \le& \frac{\Psi ^{1 - \alpha }}{\Gamma \alpha } \int _{0}^{t} \bigl\Vert \bigl[Q_{1}( \tau ,S_{n - 1}) - Q_{1}(\tau ,S_{n - 2})\bigr](t - \tau )^{\alpha - 1} \bigr\Vert \,d\tau . \end{aligned}$$

With Lipchitz condition (6), we have

7 $$ \bigl\Vert P_{1n}(t) \bigr\Vert \le \frac{\Psi ^{1 - \alpha }}{\Gamma \alpha } d_{1} \int _{0}^{t} \bigl\Vert P_{1(n - 1)}(\tau ) \bigr\Vert \,d\tau . $$

As a similar way, we obtained

8 $$ \bigl\Vert P_{in}(t) \bigr\Vert \le \frac{\Psi ^{1 - \alpha }}{\Gamma \alpha } d_{i} \int _{0}^{t} \bigl\Vert P_{i(n - 1)}(\tau ) \bigr\Vert \,d\tau ,\quad i = 2,3,\ldots,9. $$

Thus, we can write that

$$\begin{aligned}& S_{n}(t) = \sum_{j = 1}^{n} P_{1j}(t),\qquad E_{n}(t) = \sum _{j = 1}^{n} P_{2j}(t),\qquad (E_{1})_{n}(t) = \sum_{j = 1}^{n} P_{3j}(t), \\& (E_{2})_{n}(t) = \sum_{j = 1}^{n} P_{4j}(t), \\& I_{n}(t) = \sum_{j = 1}^{n} P_{5j}(t),\qquad (I_{1})_{n}(t) = \sum _{j = 1}^{n} P_{6j}(t),\qquad (I_{2})_{n}(t) = \sum_{j = 1}^{n} P_{7j}(t), \\& T_{n}(t) = \sum_{j = 1}^{n} P_{8j}(t), \qquad R_{n}(t) = \sum _{j = 1}^{n} P_{9j}(t). \end{aligned}$$

 □

The existence of a solution is given in the next theorem.

### Theorem 7.2

*A system of solutions given by the fractional SEITR model* (1) *exists if there exists* $t_{1}$ *such that*

$$ \frac{\Psi ^{1 - \alpha }}{\Gamma \alpha } t_{1}d_{i} < 1. $$

### Proof

From the recursive technique and Eq. (7) and Eq. (8) we conclude that

$$\begin{aligned}& \bigl\Vert P_{1n}(t) \bigr\Vert \le \bigl\Vert S_{n}(0) \bigr\Vert \biggl[ \frac{\Psi ^{1 - \alpha }}{\Gamma \alpha } d_{1}t \biggr]^{n}, \\& \bigl\Vert P_{2n}(t) \bigr\Vert \le \bigl\Vert E_{n}(0) \bigr\Vert \biggl[ \frac{\Psi ^{1 - \alpha }}{\Gamma \alpha } d_{2}t \biggr]^{n}, \\& \bigl\Vert P_{3n}(t) \bigr\Vert \le \bigl\Vert (E_{1})_{n}(0) \bigr\Vert \biggl[ \frac{\Psi ^{1 - \alpha }}{\Gamma \alpha } d_{3}t \biggr]^{n}, \\& \bigl\Vert P_{4n}(t) \bigr\Vert \le \bigl\Vert (E_{2})_{n}(0) \bigr\Vert \biggl[ \frac{\Psi ^{1 - \alpha }}{\Gamma \alpha } d_{4}t \biggr]^{n},\qquad \bigl\Vert P_{5n}(t) \bigr\Vert \le \bigl\Vert I_{n}(0) \bigr\Vert \biggl[ \frac{\Psi ^{1 - \alpha }}{\Gamma \alpha } d_{5}t \biggr]^{n}, \\& \bigl\Vert P_{6n}(t) \bigr\Vert \le \bigl\Vert (I_{1})_{n}(0) \bigr\Vert \biggl[ \frac{\Psi ^{1 - \alpha }}{\Gamma \alpha } d_{6}t \biggr]^{n}, \\& \bigl\Vert P_{7n}(t) \bigr\Vert \le \bigl\Vert (I_{2})_{n}(0) \bigr\Vert \biggl[ \frac{\Psi ^{1 - \alpha }}{\Gamma \alpha } d_{7}t \biggr]^{n},\qquad \bigl\Vert P_{8n}(t) \bigr\Vert \le \bigl\Vert T_{n}(0) \bigr\Vert \biggl[ \frac{\Psi ^{1 - \alpha }}{\Gamma \alpha } d_{8}t \biggr]^{n}, \\& \bigl\Vert P_{9n}(t) \bigr\Vert \le \bigl\Vert R_{n}(0) \bigr\Vert \biggl[ \frac{\chi ^{1 - \alpha }}{\Gamma \alpha } d_{9}t \biggr]^{n}. \end{aligned}$$

Thus, the system has a continuous solution. To prove that the above functions construct a solution for model (2), we assume that

$$\begin{aligned}& S(t) - S(0) = S_{n}(t) - W_{1n}(t),\qquad E(t) - E(0) = E_{n}(t) - W_{2n}(t), \\& (E_{1}) (t) - (E_{1}) (0) = (E_{1})_{n}(t) - W_{3n}(t), \\& (E_{2}) (t) - (E_{2}) (0) = (E_{2})_{n}(t) - W_{4n}(t),\qquad I(t) - I(0) = I_{n}(t) - W_{5n}(t), \\& (I_{1}) (t) - (I_{1}) (0) = (I_{1})_{n}(t) - W_{6n}(t), \\& (I_{2}) (t) - (I_{2}) (0) = (I_{2})_{n}(t) - W_{7n}(t),\qquad T(t) - T(0) = T_{n}(t) - W_{8n}(t), \\& R(t) - R(0) = R_{n}(t) - W_{9n}(t). \end{aligned}$$

So

$$ \bigl\Vert W_{1n}(t) \bigr\Vert \le \frac{\Psi ^{1 - \alpha }}{\Gamma \alpha } \int _{0}^{t} \bigl\Vert Q_{1}(\tau ,S) - Q_{1}(\tau ,S_{n - 1}) \bigr\Vert \,d\tau \le \frac{\Psi ^{1 - \alpha }}{\Gamma \alpha } d_{1} \Vert S - S_{n - 1} \Vert t. $$

By repeating the method, we obtain

$$ \bigl\Vert W_{1n}(t) \bigr\Vert \le \biggl[ \frac{\Psi ^{1 - \alpha }}{\Gamma \alpha } t \biggr]^{n + 1}d_{1}^{n + 1}h. $$

At $t_{1}$, we get

$$ \bigl\Vert W_{1n}(t) \bigr\Vert \le \biggl[ \frac{\Psi ^{1 - \alpha }}{\Gamma \alpha } t_{1} \biggr]^{n + 1}d_{1}^{n + 1}h. $$

As *n* approaches to ∞, this implies $\Vert W_{1n}(t) \Vert \to 0$. Similarly, we can obtain $\Vert W_{in}(t) \Vert \to 0$, $i = 2,3,4,5,6,7,8,9$. Hence the theorem is proved.

To prove the uniqueness of the solution, consider that the system has another solution such as $S_{\varpi } (t)$, $E_{\varpi } (t)$, $E_{1\varpi } (t)$, $E_{2\varpi } (t)$, $I_{\varpi } (t)$, $I_{1\varpi } (t)$, $I_{2\varpi } (t)$, $T_{\varpi } (t)$, and $R_{\varpi } (t)$, then we have

$$ S(t) - S_{\varpi } (t) = \frac{\Psi ^{1 - \alpha }}{\Gamma \alpha } \int _{0}^{t} \bigl(Q_{1}(\tau ,S) - Q_{1}(\tau ,S_{\varpi } ) \bigr)\,d\tau . $$

We take the norm of this equation

$$ \bigl\Vert S(t) - S_{\varpi } (t) \bigr\Vert \le \frac{\Psi ^{1 - \alpha }}{\Gamma \alpha } \int _{0}^{t} \bigl\Vert \bigl(Q_{1}( \tau ,S) - Q_{1}(\tau ,S_{\varpi } )\bigr) \bigr\Vert \,d\tau . $$

It follows from Lipschitz condition (3) that

$$ \bigl\Vert S(t) - S_{\varpi } (t) \bigr\Vert \le \frac{\Psi ^{1 - \alpha }}{\Gamma \alpha } d_{1}t \bigl\Vert S(t) - S_{\varpi } (t) \bigr\Vert . $$

Thus,

9 $$ \bigl\Vert S(t) - S_{\varpi } (t) \bigr\Vert \biggl( 1 - \frac{\Psi ^{1 - \alpha }}{ \Gamma \alpha } d_{1}t \biggr) \le 0. $$

 □

### Theorem 7.3

*The solution of model* (3) *is unique if the following condition holds*:

$$ \biggl( 1 - \frac{\Psi ^{1 - \alpha }}{\Gamma \alpha } d_{1}t \biggr) > 0. $$

### Proof

Suppose that condition (9) holds

$$ \bigl\Vert S(t) - S_{\varpi } (t) \bigr\Vert \biggl( 1 - \frac{\Psi ^{1 - \alpha }}{\Gamma \alpha } d_{1}t \biggr) \le 0. $$

Then $\Vert S(t) - S_{\varpi } (t) \Vert = 0$. Therefore, we get $S(t) = S_{\varpi } (t)$. Likewise, the same equality can be shown for *E*, $E_{1}$, $E_{2}$, *I*, $I_{1}$, $I_{2}$, *T*, and *R*. □

## Numerical results

In this section, we present the numerical results for the TB-model (3). We used the Adams–Bashforth–Moulton scheme [11]. Set $h = \frac{T}{N}$, $t_{n} = nh$, $n = 0,1,2,\ldots $ , $N \in \mathbb{Z}^{ +} $, we can write system (3) as follows:

$$\begin{aligned}& S_{n + 1} = S_{0} + \frac{h^{\alpha } \Psi ^{1 - \alpha }}{\Gamma (\alpha + 2)} \bigl[ \Lambda + a_{8}R_{n + 1}^{p} - (\lambda + \mu )S_{n + 1}^{p} \bigr] \\& \hphantom{S_{n + 1} =}{}+ \frac{h^{\alpha } \Psi ^{1 - \alpha }}{\Gamma (\alpha + 2)}\sum_{i = 0}^{n} a_{i,n + 1} \bigl[ \Lambda + a_{8}R_{i} - (\lambda + \mu )S_{i} \bigr], \\& E_{n + 1} = E_{0} + \frac{h^{\alpha } \Psi ^{1 - \alpha }}{\Gamma (\alpha + 2)} \bigl[ (1 - a_{1})\lambda \bigl(S_{n + 1}^{p} + a_{5}T_{n + 1}^{p}\bigr) - (\sigma + \mu )E_{n + 1}^{p} \bigr] \\& \hphantom{E_{n + 1} =}{}+ \frac{h^{\alpha } \Psi ^{1 - \alpha }}{\Gamma (\alpha + 2)}\sum_{i = 0}^{n} a_{i,n + 1} \bigl[ (1 - a_{1})\lambda (S_{i} + a_{5}T_{i}) - (\sigma + \mu )E_{i} \bigr], \\& (E_{1})_{n + 1} = (E_{1})_{0} + \frac{h^{\alpha } \Psi ^{1 - \alpha }}{\Gamma (\alpha + 2)} \bigl[ a_{2}\sigma E_{n + 1}^{p} + a_{3}(E_{2})_{n + 1}^{p} - a_{7}\lambda (E_{1})_{n + 1}^{p} - (r_{0} + \mu ) (E_{1})_{n + 1}^{p} \bigr] \\& \hphantom{(E_{1})_{n + 1} =}{}+ \frac{h^{\alpha } \Psi ^{1 - \alpha }}{\Gamma (\alpha + 2)}\sum_{i = 0}^{n} a_{i,n + 1} \bigl[ a_{2}\sigma E_{i} + a_{3}(E_{2})_{i} - a_{7}\lambda (E_{1})_{i} - (r_{0} + \mu ) (E_{1})_{i} \bigr], \\& (E_{2})_{n + 1} = (E_{2})_{0} + \frac{h^{\alpha } \Psi ^{1 - \alpha }}{\Gamma (\alpha + 2)} \bigl[ (1 - a_{2})\sigma E_{n + 1}^{p} - (a_{4}\lambda + k_{1} + a_{3} + \mu ) (E_{2})_{n + 1}^{p} \bigr] \\& \hphantom{(E_{2})_{n + 1} =}{}+ \frac{h^{\alpha } \Psi ^{1 - \alpha }}{\Gamma (\alpha + 2)}\sum_{i = 0}^{n} a_{i,n + 1} \bigl[ (1 - a_{2})\sigma E_{i} - (a_{4}\lambda + k_{1} + a_{3} + \mu ) (E_{2})_{i} \bigr], \\& I_{n + 1} = I_{0} + \frac{h^{\alpha } \Psi ^{1 - \alpha }}{\Gamma (\alpha + 2)} \bigl[ a_{1} \lambda \bigl(S_{n + 1}^{p} + a_{5}T_{n + 1}^{p} \bigr) + a_{7}\lambda (E_{1})_{n + 1}^{p} + (a_{4}\lambda \\& \hphantom{I_{n + 1} =}{}+ k_{1}) (E_{2})_{n + 1}^{p} - (k_{2} + \mu )I_{n + 1}^{p} \bigr] \\& \hphantom{I_{n + 1} =}{}+ \frac{h^{\alpha } \Psi ^{1 - \alpha }}{\Gamma (\alpha + 2)}\sum_{i = 0}^{n} a_{i,n + 1} \bigl[ a_{1}\lambda (S_{i} + a_{5}T_{i}) + a_{7}\lambda (E_{1})_{i} + (a_{4}\lambda + k_{1}) (E_{2})_{i} - (k_{2} + \mu )I_{i} \bigr], \\& (I_{1})_{n + 1} = (I_{1})_{0} + \frac{h^{\alpha } \Psi ^{1 - \alpha }}{\Gamma (\alpha + 2)} \bigl[ a_{6}k_{2}I_{n + 1}^{p} - (r_{1} + \mu ) (I_{1})_{n + 1}^{p} \bigr] \\& \hphantom{(I_{1})_{n + 1} =}{}+ \frac{h^{\alpha } \Psi ^{1 - \alpha }}{\Gamma (\alpha + 2)}\sum_{i = 0}^{n} a_{i,n + 1} \bigl[ a_{6}k_{2}I_{i} - (r_{1} + \mu ) (I_{1})_{i} \bigr], \\& (I_{2})_{n + 1} = (I_{2})_{0} + \frac{h^{\alpha } \Psi ^{1 - \alpha }}{\Gamma (\alpha + 2)} \bigl[ (1 - a_{6})k_{2}I_{n + 1}^{p} - (r_{2} + \mu ) (I_{2})_{n + 1}^{p} \bigr] \\& \hphantom{(I_{2})_{n + 1} =}{}+ \frac{h^{\alpha } \Psi ^{1 - \alpha }}{\Gamma (\alpha + 2)}\sum_{i = 0}^{n} a_{i,n + 1} \bigl[ (1 - a_{6})k_{2}I_{i} - (r_{2} + \mu ) (I_{2})_{i} \bigr], \\& T_{n + 1} = T_{0} + \frac{h^{\alpha } \Psi ^{1 - \alpha }}{\Gamma (\alpha + 2)} \bigl[ r_{0}(E_{1})_{n + 1}^{p} + r_{1}(I_{1})_{n + 1}^{p} + r_{2}(I_{2})_{n + 1}^{p} - (a_{1}a_{5}\lambda + r_{3} + \mu )T_{n + 1}^{p} \bigr] \\& \hphantom{T_{n + 1} =}{}+ \frac{h^{\alpha } \Psi ^{1 - \alpha }}{\Gamma (\alpha + 2)}\sum_{i = 0}^{n} a_{i,n + 1} \bigl[ r_{0}(E_{1})_{i} + r_{1}(I_{1})_{i} + r_{2}(I_{2})_{i} - (a_{1}a_{5}\lambda + r_{3} + \mu )T_{i} \bigr], \\& R_{n + 1} = R_{0} + \frac{h^{\alpha } \Psi ^{1 - \alpha }}{\Gamma (\alpha + 2)} \bigl[ r_{3}T_{n + 1}^{p} - (a_{8} + \mu )R_{n + 1}^{p} \bigr] \\& \hphantom{R_{n + 1} =}{}+ \frac{h^{\alpha } \Psi ^{1 - \alpha }}{\Gamma (\alpha + 2)}\sum _{i = 0}^{n} a_{i,n + 1} \bigl[ r_{3}T_{i} - (a_{8} + \mu )R_{i} \bigr], \end{aligned}$$

where

$$\begin{aligned}& S_{n + 1}^{p} = S_{0} + \frac{\Psi ^{1 - \alpha }}{\Gamma \alpha } \sum _{i = 0}^{n} \phi _{i,n + 1} \bigl[ \Lambda + a_{8}R_{i} - (\lambda + \mu )S_{i} \bigr], \\& E_{n + 1}^{p} = E_{0} + \frac{\Psi ^{1 - \alpha }}{\Gamma \alpha } \sum _{i = 0}^{n} \phi _{i,n + 1} \bigl[ (1 - a_{1})\lambda (S_{i} + a_{5}T_{i}) - ( \sigma + \mu )E_{i} \bigr], \\& (E_{1})_{n + 1}^{p} = (E_{1})_{0} + \frac{\Psi ^{1 - \alpha }}{\Gamma \alpha } \sum_{i = 0}^{n} \phi _{i,n + 1} \bigl[ a_{2}\sigma E_{i} + a_{3}(E_{2})_{i} - a_{7}\lambda (E_{1})_{i} - (r_{0} + \mu ) (E_{1})_{i} \bigr], \\& (E_{2})_{n + 1}^{p} = (E_{2})_{0} + \frac{\Psi ^{1 - \alpha }}{\Gamma \alpha } \sum_{i = 0}^{n} \phi _{i,n + 1} \bigl[ (1 - a_{2})\sigma E_{i} - (a_{4}\lambda + k_{1} + a_{3} + \mu ) (E_{2})_{i} \bigr], \\& I_{n + 1}^{p} = I_{0} + \frac{\Psi ^{1 - \alpha }}{\Gamma \alpha } \sum _{i = 0}^{n} \phi _{i,n + 1} \bigl[ a_{1}\lambda (S_{i} + a_{5}T_{i}) + a_{7}\lambda (E_{1})_{i} + (a_{4} \lambda + k_{1}) (E_{2})_{i} - (k_{2} + \mu )I_{i} \bigr], \\& (I_{1})_{n + 1}^{p} = (I_{1})_{0} + \frac{\Psi ^{1 - \alpha }}{\Gamma \alpha } \sum_{i = 0}^{n} \phi _{i,n + 1} \bigl[ a_{6}k_{2}I_{i} - (r_{1} + \mu ) (I_{1})_{i} \bigr], \\& (I_{2})_{n + 1}^{p} = (I_{2})_{0} + \frac{\Psi ^{1 - \alpha }}{\Gamma \alpha } \sum_{i = 0}^{n} \phi _{i,n + 1} \bigl[ (1 - a_{6})k_{2}I_{i} - (r_{2} + \mu ) (I_{2})_{i} \bigr], \\& T_{n + 1}^{p} = T_{0} + \frac{\Psi ^{1 - \alpha }}{\Gamma \alpha } \sum _{i = 0}^{n} \phi _{i,n + 1} \bigl[ r_{0}(E_{1})_{i} + r_{1}(I_{1})_{i} + r_{2}(I_{2})_{i} - (a_{1}a_{5} \lambda + r_{3} + \mu )T_{i} \bigr], \\& R_{n + 1}^{p} = R_{0} + \frac{\Psi ^{1 - \alpha }}{\Gamma \alpha } \sum _{i = 0}^{n} \phi _{i,n + 1} \bigl[ r_{3}T_{i} - (a_{8} + \mu )R_{i} \bigr], \end{aligned}$$

in which

$$ a_{i,n + 1} = \textstyle\begin{cases} n^{\eta _{j} + 1} - (n - \alpha _{j})(n + 1)^{\alpha _{j}};&i = 0, \\ (n - i + 2)^{\alpha _{j} + 1} + (n - i)^{\alpha _{j} + 1} - 2(n - i + 1)^{\alpha _{j} + 1}; &1 \le i \le n, \\ 1;&i = n + 1 \end{cases} $$

and

$$ \phi _{i,n + 1} = \frac{h^{\alpha _{j}}}{\alpha _{j}}\bigl((n - i + 1)^{\alpha _{j}} - (n - i)^{\alpha _{j}}\bigr);\quad 0 \le i \le n,\text{and } j = 1,2,3,4,5. $$

## Numerical simulations

The following are the graphical simulations for the fractional orders $\alpha = 0.5,0.6,0.7,0.8, 0.9,0.99,1$.

**Figure 5:**
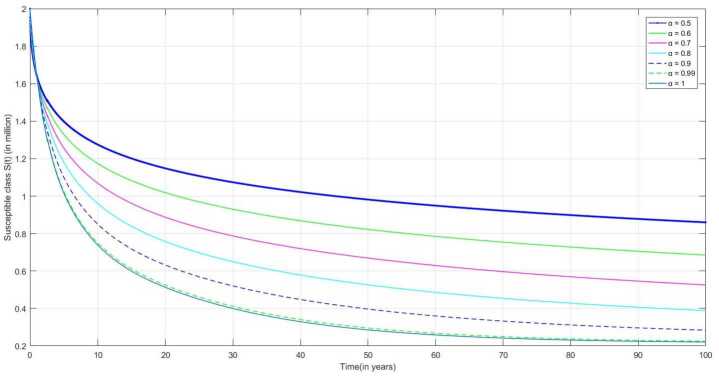
Number of susceptible individuals $S(t)$ v/s time (t)

In many countries, the BCG vaccine is given to a newly born baby. It can be observed from the figure that the number of susceptible individuals decreases over time as people are immunized through BCG vaccination. In Fig. 5, we can observe that the number of susceptible individuals decreases over time.

**Figure 6:**
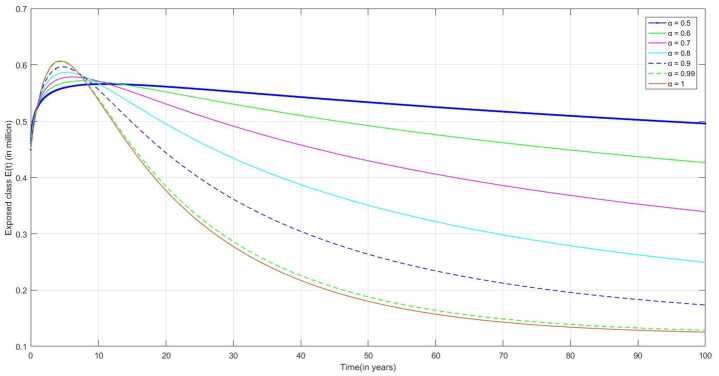
Number of new latently infected individuals $E(t)$ v/s time (t)

In Fig. 6, due to vaccination, the number of exposed individuals to TB also decreases over time. However, many people are still reported as TB-infected with different compartments. As NLI individuals do not have any symptoms and feel sick, they are not spreaders of TB bacteria to others; but can be identified using skin or blood tests. We can also observe from Fig. 3 that initially the number of NLI individuals increases, and then due to the decrease in the number of susceptible individuals concerning time, the number of NLI individuals decreases slowly over time.

**Figure 7:**
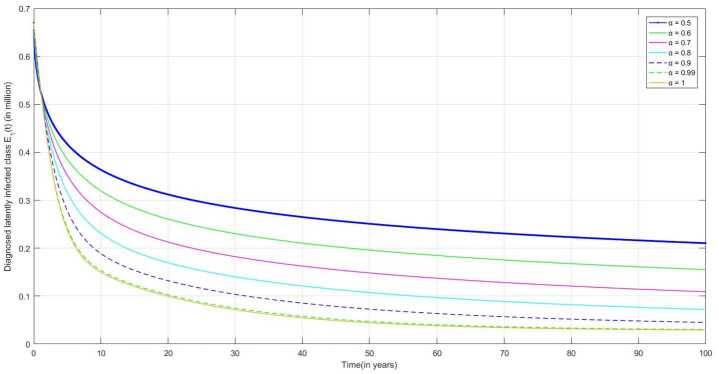
Number of diagnosed latently infected Individuals $E_{1}(t)$ v/s time (t)

Because of awareness regarding TB infection, people are taking precautions in the initial compartments, and if diagnosed as TB-infected, the person becomes diagnosed latently infected (DLI) and then starts taking treatment. In Fig. 7, we can observe that the number of DLI individuals is increasing slowly over time as NLI individuals are diagnosed positive.

**Figure 8:**
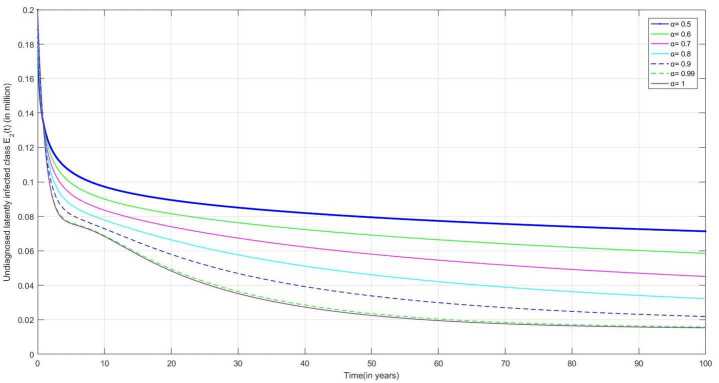
Number of undiagnosed latently infected individuals $E_{2}(t)$ v/s time (t)

In Fig. 8, as discussed earlier, from the symptoms of NLI-TB we can observe that the number of ULI individuals is increasing over time. As the duration of the treatment is long, some people stop taking treatment as they feel better and hence do not recover completely, which may convert them into actively infected and spread the infection to other people.

**Figure 9:**
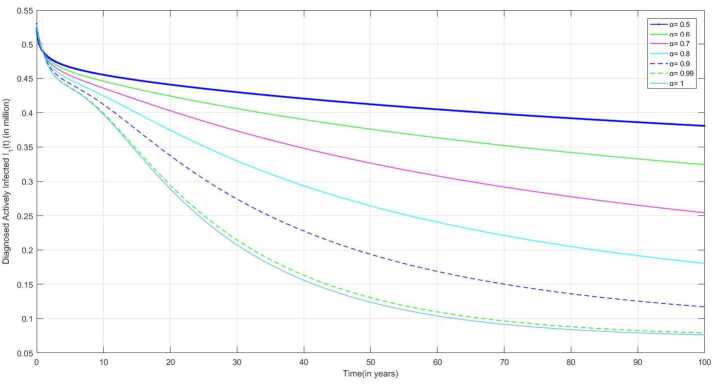
Number of diagnosed individuals as actively infected with prompt treatment $I_{1}(t)$ v/s time (t)

In Fig. 9, due to the awareness about TB disease, infected people start taking medication immediately. We can observe that the number of DAI with prompt treatment increases over the time after diagnosed as positively infected.

**Figure 10:**
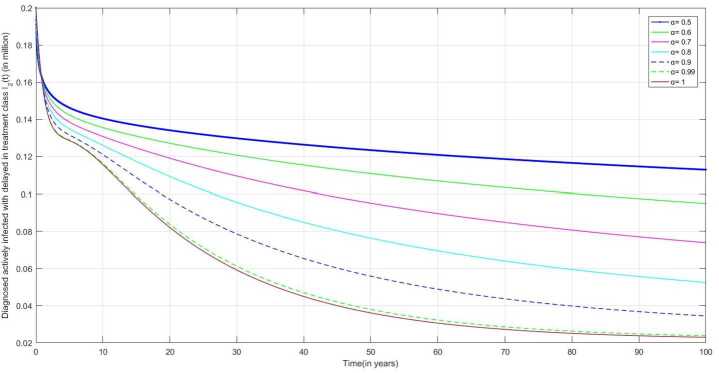
Number of individual diagnosed as actively infected with delay treatment individuals $I_{2}(t)$ v/s time (t)

The individual who is not diagnosed in the initial compartment may become actively infected and also spread the infection to others. People having symptoms of TB infection but not diagnosed as TB-infected and people diagnosed as positive but not starting treatment may move to treatment class after reaching more severe conditions. In Fig. 10, we can observe that the infection of some people who are diagnosed as infected becomes quite severe, and then they start taking medication, due to which the number of DAI individuals with delay in treatment increases after some time.

**Figure 11:**
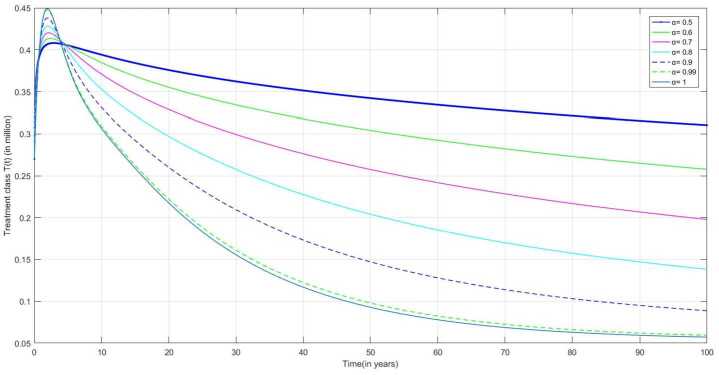
Number of treated individuals $T(t)$ v/s time (t)

Figure 11 represents the number of individuals under the treatment after diagnosed as infected. This includes the number of individuals in DLI, DLI with prompt treatment and DLI with delay treatment. Perhaps, during the treatment some people may become drug-resistant and can be classified as MDR and XDR. As in all compartments, people are getting TB infection, the number of infected individuals increases initially and then decreases over time.

**Figure 12:**
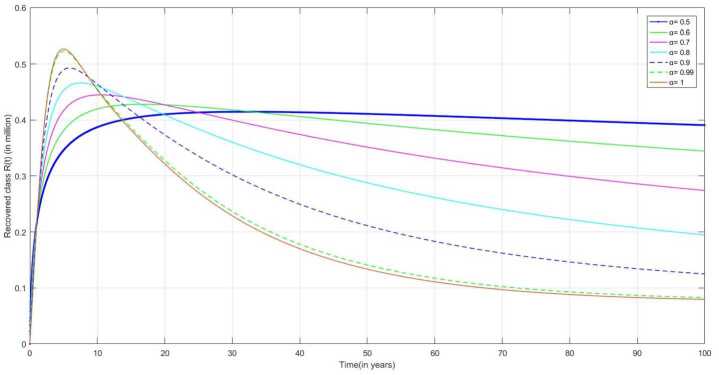
Number of recovered/removed or stop treatment individuals $R(t)$ v/s time (t)

Figure 12 represents the number of recovered/removed individuals. The duration of medication or treatment takes longer time for TB. So, after getting partial recovery, some people stop medication or treatment. The individual recovered from this stage may become susceptible for the infection and may have chance of getting infected again.

**Figure 13:**
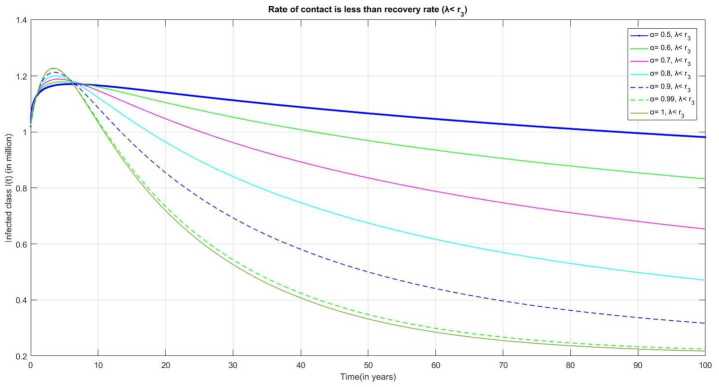
Number of infected individual $I(t)$ over the time(t) when $\lambda < r_{3}$

From Fig. 13 we observe that if the contact rate *λ* of an infected individual is considered as lower than the recovery rate $r_{3}$, the number of infected people rises at first, then drops significantly.

**Figure 14:**
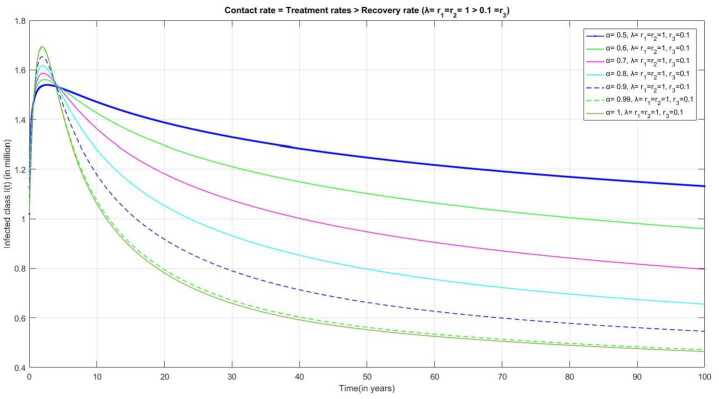
Number of infected individuals $I(t)$ over the time(t) when $\lambda = r_{1} = r_{2} = 1$ and $r_{3} = 0.1$

Figure 14 represents the behavior of the infected individual when the contact rate *λ*, treatment rates $r_{1}$ and $r_{2}$ are considered as 1 and the recovery rate as $r_{3} = 0.1$, then we can observe a rapid increase in the number of infected individuals and increased burden on treatment class. Moreover, the recovery rate is lower than the contact rate and the treatment rate, which decreases the number of infected individuals due to the mortality through infection.

**Figure 15:**
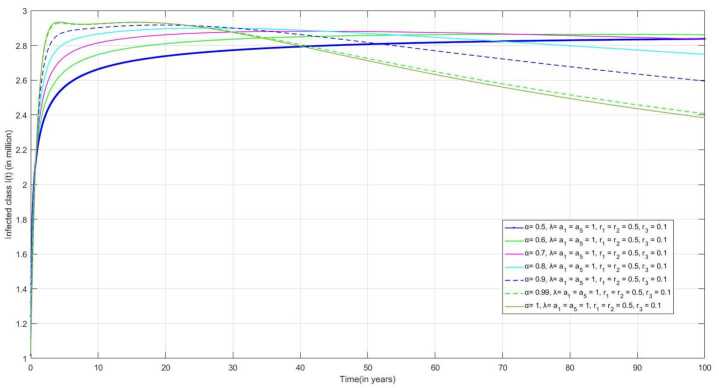
Number of infected individuals $I(t)$ over the time(t) when $\lambda = a_{1} = a_{5} = 1$, $r_{1} = r_{2} = 0.5$,and $r_{3} = 0.1$

For Fig. 15, we have considered the values of the contact rate $\lambda = 1$, the rate at which an individual from the treatment class joins the infected class $a_{1} = a_{5} = 1$, the treatment rates $r_{1}$ and $r_{2}$ are considered as 0.5 and the recovery rate as $r_{3} = 0.1$. As the recovery rate is very low in comparison to other rates, the number of infected individuals in the infected class increases promptly.

## Conclusion

In this paper, a dynamical fractional-order mathematical model in the Caputo sense is proposed for the behavioral investigation of TB infection in India. Using the nonlinear least square algorithm, we estimated the parameters used in this model using TB-infected cases reported in India from the year 2000 to 2020, which shows that the model curve provides a good fit to the real data. Moreover, the future trend of the model curve provides indication to the decision maker or policy maker for better devising disease prevention and control measures. Further, we present the endemic equilibria, locally asymptotically stable, and the TB infection-free equilibrium in terms of the basic reproduction number. The value of the basic reproduction is obtained as $\Re _{0} = 1.7307$ demonstrating the importance of appropriate control and prevention strategies. The existence and uniqueness of the approximate solution for the model is derived using the generalized Adams–Bashforth–Moulton method in the numerical results. The graphical representation is provided to demonstrate the flow of all compartments for $\alpha = 0.5,0.6,0.7,0.8,0.9,0.99,1$. The integer order model represents the rate of infection at order 1, but fails to provide any information between order 0 and 1, which is important to identify the initial behavior of the infection. However, our model represents the fractional rate of infection at different order between 0 and 1 to capture the significant information and analyze the intricate dynamics of the infection of tuberculosis for better apprehension, which can be observed in all the above graphical representation. Tuberculosis infection is curable, but still the infection is transmitting rapidly between the community. Also, from the real data of TB infection in India [3] we can observe that the confirmed cases of TB infection decreased during the COVID-19 lockdown in India from March 2020 to December 2020. During the lockdown, people were using masks, sanitizer, maintained social distancing, and followed COVID-19 guidelines; and this action plan played a vital role inpreventing TB infection among the population of India. Further, we can extend this model by considering the regimen changed compartment or the exempted compartment for non-evaluate individual or both separately.

## Data Availability

The data used in this manuscript are available on the following websites: (1) https://tbfacts.org/tb-statistics-india/; (2) https://tbcindia.gov.in; (3) https://pib.gov.in/PressReleseDetail.aspx?PRID=1606209; (4) https://www.worldometers. info/world-population/india-population/#:~:text=India%202020%20population%20is%20estimated,(and%20 dependencies)%20by%20population; (5) https://www.thehindu.com/data/data-tb-deaths-on-a-seven-year-high-as-case-notifications-and-outpatient-visits-dipped-in-2020/article37098076.ece.
